# Brain not processing: is finding a role for BNP in sepsis like fitting a square peg into a round hole?

**DOI:** 10.1186/cc13960

**Published:** 2014-07-01

**Authors:** Anthony S McLean, Stephen J Huang

**Affiliations:** 1 Department of Intensive Care Medicine, Nepean Hospital, University of Sydney, Parker Street, Sydney, NSW 2750, Australia

## Abstract

Since its introduction to the intensive care setting a decade ago, B-type natriuretic peptide has been the focus of studies in different areas (in particular, sepsis). With this biomarker, as with many newly identified biomarkers, its diagnostic performance was pursued initially and then its ability to predict outcomes. Despite all the efforts, results have not been consistent and the applications of B-type natriuretic peptide in the intensive care setting remain by and large academic. Will such studies one day become clinical practice? Or are we too obsessed with finding a place for every biomarker?

## Introduction

The 108-amino acid prohormone B-type natriuretic peptide (BNP) is released into the circulation during hemodynamic stress and, once in the circulation, is cleaved by a circulating endopeptidase, corin, into the inactive 76-amino acid NT-proBNP (N-terminal of the prohormone brain natriuretic peptide) and biologically active 32-amino acid BNP. BNP causes arterial vasodilatation, diuresis, and natriuresis, which result in a lowering of both preload and afterload. BNP is cleared via internalization by cells that express BNP receptors while renal clearance is the main mechanism for NT-proBNP. This difference accounts for the difference in plasma concentration half-lives and is likely to be responsible, in part, for delayed clearance in patients with sepsis. The finding of elevated plasma levels of BNP or NT-proBNP in sepsis over a decade ago has resulted in an ongoing search for a meaningful role of the hormone in clinical practice. The article by Papanikolaou and colleagues [1] in this issue of *Critical Care* represents the latest search in this area.

## B-type natriuretic peptide in sepsis

Although is it is not unexpected that patients with sepsis-induced cardiomyopathy would exhibit elevated plasma BNP and NT-proBNP levels, such elevations are also found to be elevated in sepsis in the absence of overt cardiac dysfunction.

Though BNP has proven to be a useful biomarker in heart failure, a diagnostic role for BNP in the patient with sepsis has failed to materialize. This is because multiple factors cause an elevation of plasma BNP, factors commonly accompanying the presence of severe sepsis, such as fluid perturbations and ongoing treatment. There may be a role in select situations; a recent publication demonstrated that elevation of NT-proBNP in patients with burns has both high sensitivity and specificity for superadded infection [2].

When a biomarker is found to be unsuitable as an accurate diagnostic marker, the next step is to evaluate a prognostic role or monitoring therapy or both. The literature is now replete with studies. As Papanikolaou and colleagues point out, although BNP has been promoted as a useful prognosticator for septic shock, the results were inconsistent: some studies reported the measurement on day 2 as useful, some on day 3, and so on [3,4]. On the other hand, our previous study [5] and a later one by Rudiger and colleagues [6] did not find BNP to be useful to predict mortality in these patients.

Does the study by Papanikolaou and colleagues, who looked at the mechanism of BNP elevation in sepsis, elucidate this ongoing debate? It is a single-center study involving 42 patients over the course of 3 years and it used pulmonary artery catherization and echocardiography for hemodynamic assessment, and plasma BNP levels were performed daily for 5 days. The study can be described as an exploratory type and has a number of interesting findings: (a) the severity of illness rather than sepsis-induced myocardial depression was the main determinant of BNP levels; (b) the baseline left ventricular ejection fractions (LVEFs) in the survivors were similar to those in non-survivors; (c) BNP was not independently associated with reduced LVEF; (d) in septic shock, there were no differences in daily BNP levels between the survivors and non-survivors; and (e) in patients with critical sepsis, there was marginal evidence (*P* = 0.049) that the BNP on day 1 was higher in non-survivors. The difficulty with the last discovery is the uncertainty of whether this was a mere coincidence by chance from multiple comparisons. The authors also found that the rate of decline of BNP over the 5-day monitoring period was faster in survivors, and introduced two ‘clinical markers’ for predicting 28-day mortality: (a) based on the rate of decline, BNP level failed to fall below 500 pg/mL in any of the 5 days of monitoring; and (b) BNP/central venous pressure ratio was more than 126 pg/mL per mmHg on day 2. Although these two markers look promising, we should approach the results with caution: the first ‘clinical marker’ suffers from less than adequate specificity (62%), whereas the predictive performance of the second can be described as moderate at best. The results of this study, like those of many other exploratory studies, need to be validated by a larger independent cohort.

## Conclusions

In the face of conflicting results as to whether plasma BNP and NT-proBNP levels predict outcome, the question is raised as to why we as critical care physicians are so dogged in our determination to fit BNP or NT-proBNP plasma levels into our management algorithms. Is it because it is increased in sepsis and measuring it is easy? Given that the critically ill septic population is so heterogeneous in nature, it is unlikely that a sound and robust utilization of the test will ever be identified. Even if there is one, applying such prognostic results to an individual patient will be another challenge. Perhaps it says more about our lack of cognitive reasoning than anything else and indicates that our energies should be directed elsewhere in attempting to identify a biomarker that will assist the clinician in managing this group of patients with such a high mortality.

## Abbreviations

BNP: B-type natriuretic peptide; LVEF: Left ventricular ejection fraction; NT-proBNP: N-terminal of the prohormone brain natriuretic peptide.

## Competing interests

The authors declare that they have no competing interests.
